# Transcriptomic analysis of the innate immune response to *in vitro* transfection of plasmid DNA

**DOI:** 10.1016/j.omtn.2022.11.025

**Published:** 2022-12-05

**Authors:** Eric Warga, Jared Anderson, Matthew Tucker, Emily Harris, Jacob Elmer

**Affiliations:** 1 Department of Chemical and Biological Engineering, Villanova University, White Hall, Room 119, 800 Lancaster Avenue, Villanova, PA 19085, USA

**Keywords:** MT: Oligonucleotides: Therapies and applications, plasmid, innate immune response, cytokines, cytokine-stimulated genes, IFI16, STING, MYD88, IKBKE

## Abstract

The innate immune response to cytosolic DNA is intended to protect the host from viral infections, but it can also inhibit the delivery and expression of therapeutic transgenes in gene and cell therapies. The goal of this work was to use mRNA sequencing to identify genes that may influence transfection efficiency in four different cell types (PC-3, Jurkat, HEK-293T, and primary T cells). The highest transfection efficiency was observed in HEK-293T cells, which upregulated only 142 genes with no known antiviral functions after transfection with lipofectamine. Lipofection upregulated 1,057 cytokine-stimulated genes (CSGs) in PC-3 cells, which exhibited a significantly lower transfection efficiency. However, when PC-3 cells were transfected in serum-containing media or electroporated, the observed transfection efficiencies were significantly higher while the expression levels of cytokines and CSGs decreased. In contrast, lipofection of Jurkat and primary T cells only upregulated a few genes, but several of the antiviral CSGs that were absent in HEK-293T cells and upregulated in PC-3 cells were observed to be constitutively expressed in T cells, which may explain the relatively low Lipofection efficiencies observed with T cells (8%–21% GFP^+^). Indeed, overexpression of one CSG (IFI16) significantly decreased transfection efficiency in HEK-293T cells.

## Graphical abstract

**Figure:**
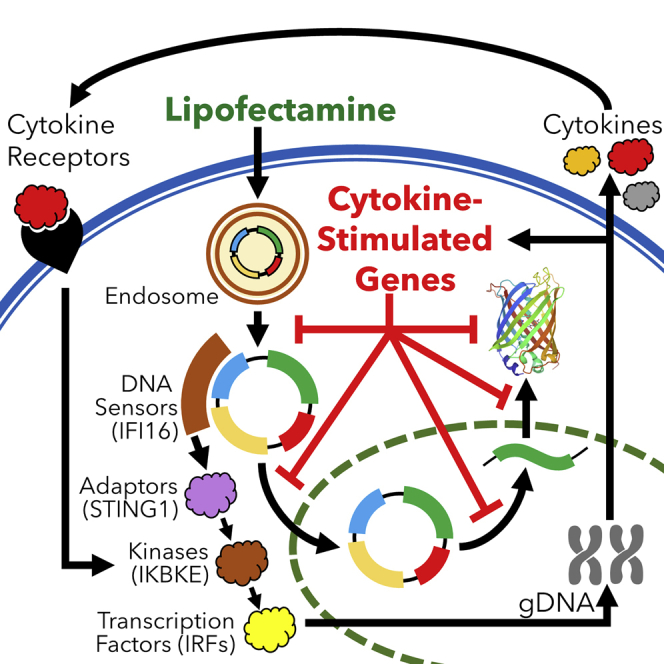

## Teaser

mRNA sequencing reveals that key proteins involved in the innate immune response to cytosolic DNA are absent in cells that are efficiently transfected with lipofectamine (HEK-293T), but highly upregulated after transfection or constitutively expressed in cell lines that are more difficult to transfect (PC-3 and T cells, respectively).

## Introduction

In recent years, multiple gene therapy treatments using adeno-associated viruses (AAVs) and lentiviruses (LVs) have been shown to effectively treat a variety of diseases in the clinic.^1^ For example, AAV is used to deliver a functional copy of the RPE65 gene to patients with Leber’s congenital amaurosis in Luxturna,^2^ while Zolgensma uses an AAV to deliver the SMN1 gene to patients with spinal muscular atrophy. Likewise, delivery of the chimeric antigen receptor to T cells is done with an LV in five different CAR-T cell therapies for B cell lymphomas (Kymriah, Yescarta, Tecartus, Abecma, and Carvykti).^3^^,^^4^^,^^5^ Several other promising gene therapies for hemophilia, Duchenne’s muscular dystrophy, immunodeficiency, and Pompe disease are also progressing through clinical trials.^6^^,^^7^^,^^8^^,^^9^ All of these therapies have demonstrated that gene delivery can be a lifesaving treatment for patients where other treatments have failed.^4^ However, several issues have arisen in regards to LV and AAV vehicles, including high treatment costs (e.g., $2M for Zolgensma), safety concerns, and low transduction efficiencies in some patients.^2^^,^^4^^,^^10^^,^^11^^,^^12^^,^^13^^,^^14^^,^^15^^,^^16^

These issues with viral gene delivery vehicles have motivated a growing demand for safer and less expensive nonviral gene delivery methods. Several nonviral vehicles and methods have been developed, including cationic lipids, polymers, lipid/polymer hybrids, nanoparticles, and electroporation.^1^^,^^17^^,^^18^^,^^19^ However, while these methods are generally less expensive and potentially safer than AAVs and LVs, they tend to provide lower transfection efficiencies than viruses.^20^^,^^21^ Consequently, improving the efficiency of nonviral transgene delivery and expression is an important and highly active area of research.

**Figure 1:**
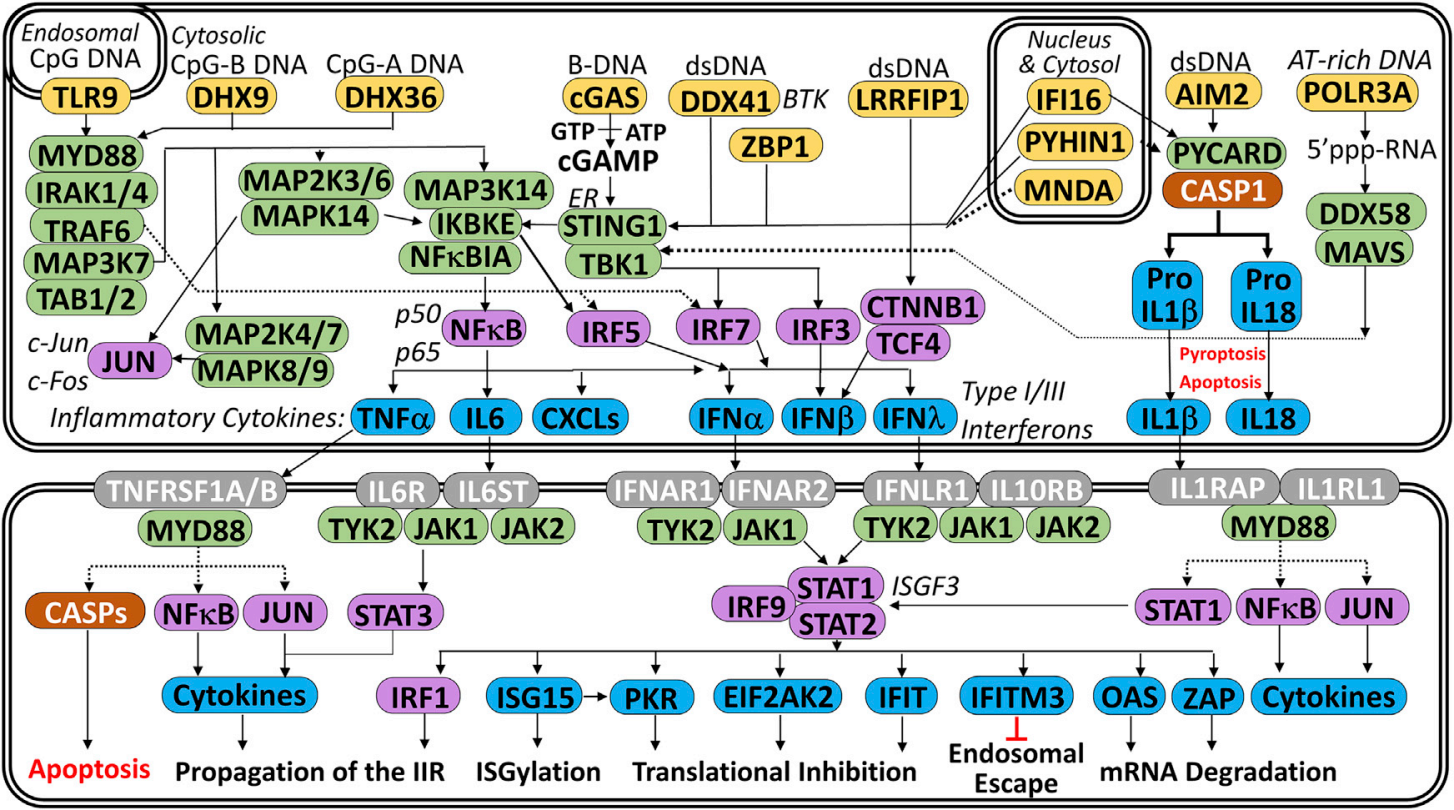
Overview of the innate immune response to foreign DNA pDNA can be recognized by several DNA sensors (yellow), which trigger a cascade of kinases (green) and transcription factors (purple) that culminates in the expression of cytokines that are secreted and bind to cognate receptors (gray) that induce the expression of cytokine stimulated genes (blue) that can trigger apoptosis or inhibit transgene expression in a variety of ways.

While it is possible that gene delivery is a limiting step for some nonviral gene delivery vehicles, another factor that could significantly inhibit transgene expression in both viral and nonviral systems is the innate immune response, which is shown in Figure 1. For example, transgenes can be detected by endosomal, cytosolic, or nuclear DNA sensors, which then use adaptor proteins like STING to trigger a signaling cascade of kinases and transcription factors that culminates in the activation of cytokines (e.g., interferon λ). These cytokines activate additional pathways that induce the expression of cytokine-stimulated genes (CSGs) like IFIT1 and OAS1 that can directly inhibit the delivery and expression of viral and non-viral transgenes.^22^^,^^23^^,^^24^ For example, IFI16 has been shown to decrease plasmid-driven transgene expression by directly binding and blocking viral promoters.^25^^,^^26^ In addition, IFI16 is also a cytosolic/nuclear double-stranded DNA (dsDNA) sensor that amplifies the innate immune response by activating STING, which then activates IRF3.^27^^,^^28^^,^^29^

While many studies have focused on specific components of the innate immune response to plasmid DNA and nonviral gene delivery, our overall knowledge of the transcriptomic profile of different cell types following transfection is still incomplete. In this study, mRNA sequencing was used to elucidate the innate immune response to plasmid DNA at the transcriptome level in a panel of four cell lines (HEK-293T, PC-3, Jurkat, and primary T cells) with varying transfection efficiencies to identify host cell genes that may inhibit transfection. Each of these cell lines was chosen because of their clinical and industrial relevance. HEK-293T is one of the most commonly used cell lines in biomanufacturing, since it has been transformed with the SV40 large T antigen to create a cell line that is relatively easy to culture and transfect.^30^^,^^31^^,^^32^^,^^33^ The transfection efficiency of the PC-3 cell line tends to be lower, but prostate cancer is one of the most frequently occurring cancer forms in men, so understanding the response of prostate cancer (PC-3) cells to gene delivery may be useful in the development of gene therapies for cancer.^34^ Likewise, two types of T cells (leukemic Jurkat T cells and primary T cells) were included in this study because gene delivery to T cells is an essential step in CAR-T cell therapies for multiple types of leukemia.^35^ Finally, since PC-3 cells have a particularly potent innate immune response to plasmid DNA (pDNA), the effects of serum, pDNA concentration, and electroporation were also studied in this cell line.

## Results

### Transfection efficiency

**Figure 2:**
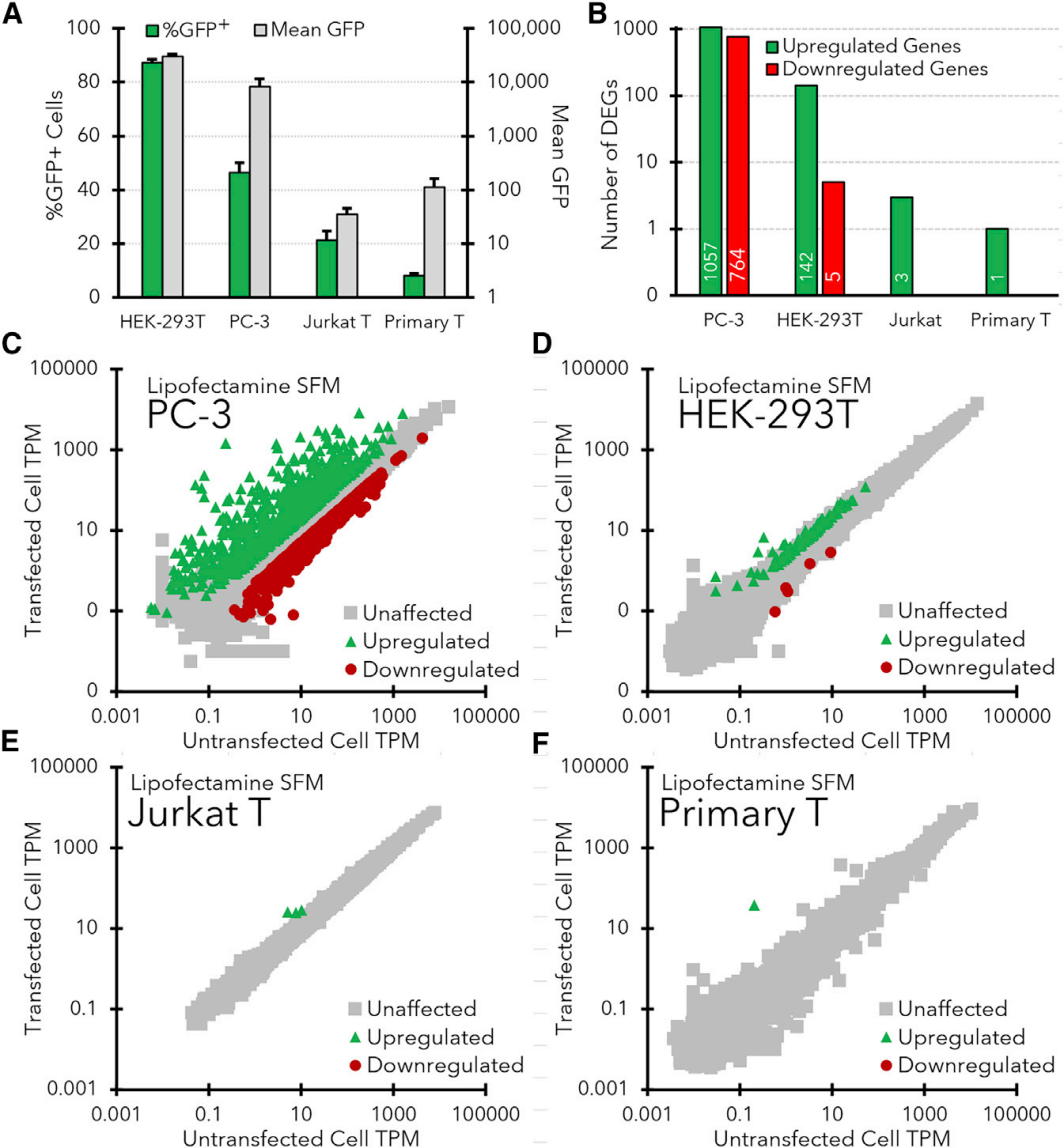
Differences in transfection efficiency and transcriptomes between cell lines transfected with lipofectamine LTX in SFMd (A) Transfection efficiency (%GFP^+^ cells) and transgene expression levels (mean GFP) for each cell line at 24 h after transfection with lipofectamine LTX and pEF-GFP pDNA in SFM. Representative flow cytometry histograms and fluorescent microscopy images for each cell line are also shown in Figure S1. Error bars indicate standard deviation of the triplicate samples. (B) Total number of upregulated and downregulated DEGs observed in each cell line after lipofection in SFM. DEGs were defined as having at least a 2-fold change in TPM that was statistically significant (p_adj_ < 0.05) over the course of three independent experiments. (C–F) Gene expression levels (TPMs) in HEK-293T cells (C), PC-3 cells (D), Jurkat T cells (E), and primary CD3^+^ T cells (F) that were either transfected with lipofectamine LTX (*y* axis) or not transfected (*x* axis). All TPM values are averaged from three separate transfections and corresponding mRNA-sequencing experiments. Green triangles indicate genes that were significantly upregulated in transfected cells (p_adj_ < 0.05), red circles indicate downregulated genes (p_adj_ < 0.05), and gray squares represent unaffected genes.

The transfection efficiencies (percent green fluorescent protein-positive [%GFP^+^] cells) and transgene (GFP) expression levels for each cell line (HEK-293T, PC-3, Jurkat, and primary T cells) with lipofectamine are shown in Figure 2A. Lipofection of pDNA into HEK-293T cells in serum-free media (SFM) provides a relatively high transfection efficiency (87.3 ± 1.2% GFP^+^ cells; measured at 24 h after transfection), while progressively lower transfection efficiencies were observed for PC-3 (46.3 ± 3.7% GFP^+^ cells) and Jurkat T cells (21.2 ± 3.4%). Finally, despite optimization of a lipofection protocol in serum-free X-VIVO 15 media, the maximum lipofection efficiency obtained for primary T cells was 8.1 ± 0.8%.

A similar downward trend was also observed in GFP expression levels between the cell lines (Figure 2A, gray bars). While GFP was expressed at high levels in HEK-293T cells (mean GFP = 30,266 ± 2,715) that were brightly fluorescent (Figure S1C), GFP levels were significantly lower in PC-3 cells (mean GFP = 8,332 ± 3,239) and extremely low in Jurkat and CD3^+^ primary T cells (mean GFP = 35 ± 11 & 112 ± 50, respectively). Fluorescent microscopy images shown in the supplemental information (Figures S1C–S1F) concur with these measurements, showing a decrease in the brightness and number of fluorescent cells between the HEK-293T cells and the other cell lines.

To determine if the difference in transfection efficiencies between the cell lines was due to a difference in the amount of transgene being delivered to the nuclei of each cell type, quantitative PCR (qPCR) analyses were also conducted to measure plasmid copy numbers (normalized to the GAPDH gene) after lipofection of each cell line (Figure S1G). While the lowest plasmid copy number was observed in the Jurkat T cells (20 ± 3), the plasmid copy number in HEK-293T cells was only slightly higher (50 ± 7), whereas the plasmid copy number in PC-3 cells was much higher (941 ± 168). Therefore, there was no clear correlation between the amount of pDNA delivered and the transfection efficiency of each cell type.

### Differential gene expression

The effects of lipofection on the transcriptomes of the four cell lines are illustrated in Figure 2. A graphical summary of the number of genes that were differentially expressed between the untransfected controls and transfected samples for each cell line is shown in Figure 2B, while complete lists of the differentially expressed genes (DEGs) are included in the supplemental information (Table S8). Figure 2 also shows specific transcripts per million (TPM) plots for PC-3 (Figure 2C), HEK-293T (Figure 2D), Jurkat T cells (Figure 2E), and primary T cells (Figure 2F).

Overall, PC-3 cells exhibited the strongest innate immune response, in which 1,057 genes were upregulated and 764 genes were downregulated after lipofection in SFM (Figures 2B and 2C). In contrast, the HEK-293T cells that exhibited a high transfection efficiency upregulated only a small number of genes (n = 142), with only 10 genes that were upregulated more than 4-fold (Figure 2D). Likewise, the number of downregulated genes in HEK-293T cells was also very low (n = 5). Both types of T cells had nearly negligible responses to lipofection (Figure 2E/F), with only three significantly upregulated genes in Jurkat (MT1E, MT1F, and TMEM238) and a single upregulated gene in primary T cells (MT1H). No genes were significantly downregulated in either type of T cell after lipofection.

### Validation of NGS with rt^2^PCR and ELISA

A small set of upregulated genes that were detected in PC-3 cells via mRNA sequencing were also validated using rt^2^PCR and ELISA. Figure S3 shows that while there were some differences in the magnitude of upregulation observed with mRNA-sequencing (blue bars) and rt^2^PCR (green bars), seven different genes (interferon [IFN]B1, IFNL1/2/3, CXCL10/11, and CASP1) that were observed to be upregulated in mRNA sequencing experiments were also found to be significantly upregulated in rt^2^PCR assays, thereby reinforcing the mRNA sequencing results.

**Figure 3:**
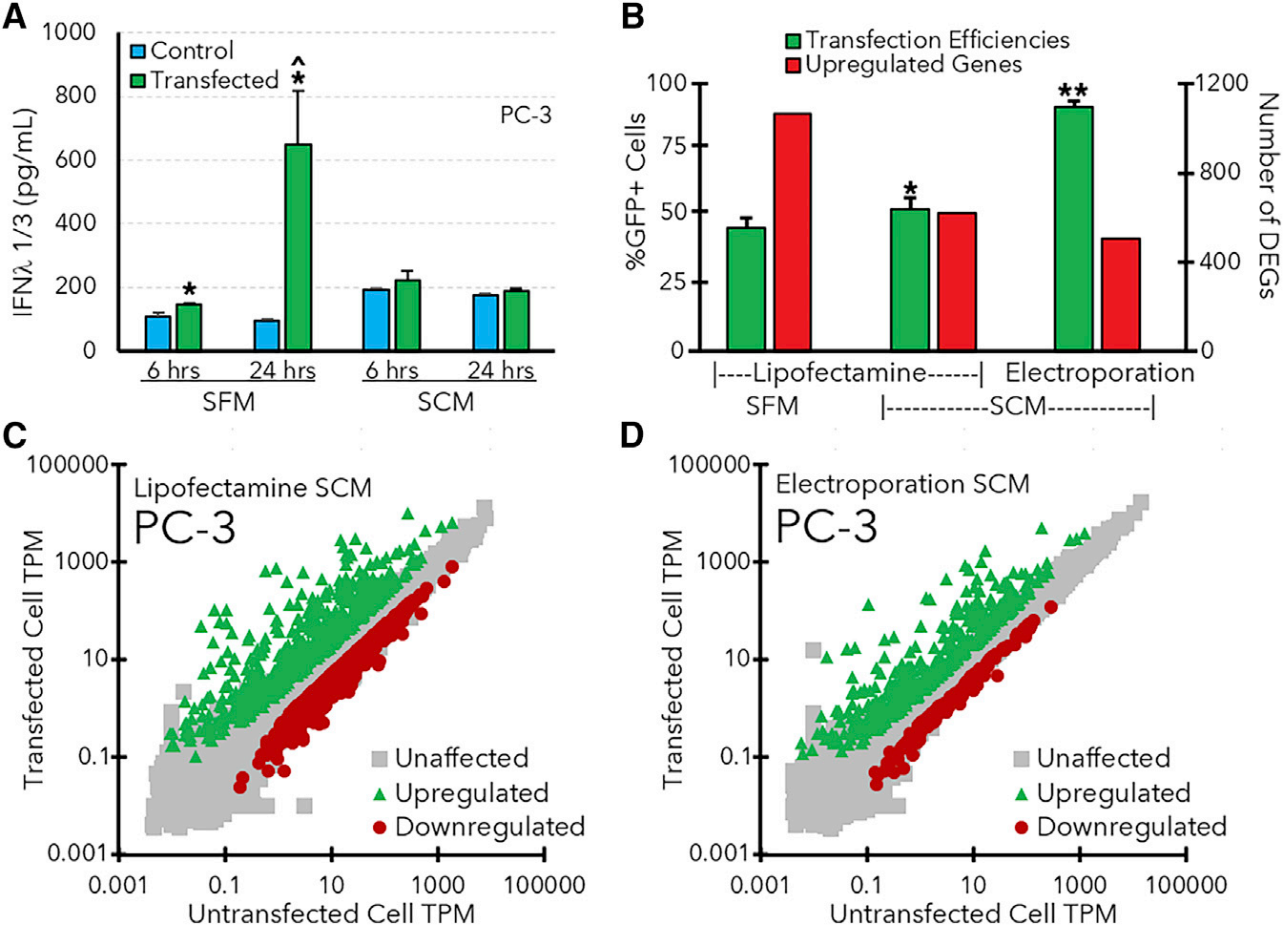
Effects of serum and electroporation on transfection efficiency and the PC-3 transcriptome (A) Effects of serum on IFN λ1/3 expression in PC-3 cells. Asterisks (∗) indicate significant (p < 0.05 as determined by Student’s t-test) increases in IFNλ levels after transfection relative to untransfected control cells, while carets (ˆ) indicate significant increases between ELISA measurements at 6 and 24 h after transfection. (B) Transfection efficiencies (green bars, left axis) and the number of significantly upregulated genes (red bars, right axis) observed after transfecting PC-3 cells with lipofectamine in SFM or SCM or electroporation in SCM. Asterisks indicate significant differences in transfection efficiency (∗significantly higher than lipofection in SFM; ∗∗significantly higher than lipofection in SFM and SCM, p < 0.05 as determined by Student’s t-test). (C/D) Gene expression levels (TPM) for PC-3 cells that were lipofected (C) or electroporated (D) in SCM. All TPM values are averaged from three separate transfections and corresponding mRNA-sequencing experiments. Green triangles indicate genes that were significantly upregulated in transfected cells, red circles indicate downregulated genes, and gray squares represent unaffected genes. All error bars indicate the standard deviation of the measurements for each bar.

Likewise, an increase in IFNλ1/3 secretion in PC-3 cells was also verified with ELISA (Figure 3A). It is worth noting that the antibody used in the ELISA assay binds to both IFNλ1 and IFNλ3, but significant increases in both IFNλ1 and IFNλ3 expression levels were detected in PC-3 cells at 6 and 24 h after lipofection in SFM (Figure 3A). IFNλ1/3 levels were also significantly higher at 24 h after lipofection than 6 h after transfection. It is also worth mentioning that other studies have used ELISA and other methods to directly show that several cytokines and chemokines, such as tumor necrosis factor (TNF)-α, interleukin (IL)-6, and IL-1β, were upregulated after dsDNA transfection in rat substantia nigra and mesangial cells, which aligns with the mRNA sequencing results shown in Figure 2.^36^^,^^37^ However, when PC-3 cells were lipofected in serum-containing media (SCM) instead of SFM, there was no significant increase in IFNλ1/3 expression levels between transfected and untransfected controls.

### Comparison of transfection methods in PC-3 cells

The observation that IFNλ1/3 levels did not increase after lipofection of PC-3 cells in SCM (Figure 3A) motivated us to further investigate the effects of serum on the transfection efficiency and transcriptome of PC-3 cells.

In addition, since electroporation is a popular nonviral gene delivery method that can provide relatively high transfection efficiencies, we also performed mRNA sequencing experiments on electroporated PC-3 cells. Figure 3B shows that electroporation (in SCM) provided a much higher transfection efficiency (92 ± 2% GFP^+^ cells) in PC-3 cells at 24 h after electroporation. Likewise, the presence of serum during lipofection also provided a slight, yet significant, increase in the transfection efficiency of the PC-3 cells (53 ± 4% GFP^+^ cells). In contrast, lipofection of Jurkat and primary T cells in SCM did not significantly increase transfection efficiency (data not shown).

Subsequent mRNA sequencing experiments revealed that the presence of serum during lipofection of PC-3 cells decreased the number of genes that were significantly upregulated (n = 619) (Figures 3B and 3C) compared with SFM (n = 1,057). Likewise, electroporation also resulted in a substantial decrease in the number of upregulated and downregulated genes (n = 533 and n = 138) (Figures 3B and 3D) compared with lipofection in both SFM and SCM. Both methods also led to an approximate 3-fold reduction in IFNλ TPM values relative to the PC-3 cells transfected in SFM, which correlates with the ELISA results shown in Figure 3A. A complete list of the DEGs identified in these experiments and their gene expression levels (TPM) is shown in a worksheet in the supplemental information (Table S8).

A potential explanation for the greater lipofection efficiency of PC-3 cells in SCM is that components in the serum may bind to the liposomes and decrease the amount of pDNA delivered to the cells. It may initially seem paradoxical to suggest that decreasing the amount of pDNA delivered to the cells could increase transfection efficiency, but the qPCR results in Figure S1G show that the pDNA copy number in PC-3 cells is 19-fold higher than the pDNA copy number in the HEK-293T cells, which have a higher transfection efficiency. Therefore, our initial lipoplex formulation may have delivered an unnecessary excess of pDNA, which could exacerbate the particularly potent innate immune response to foreign DNA in PC-3 cells. To test this hypothesis, we conducted a series of transfections in 24-well plates over a decreasing range of pDNA concentrations (2, 1, 0.5, 0.25, and 0.1 μg pDNA/well of 50,000 PC-3 cells). Figures S4 and S5 show that transfection efficiency significantly increased as the amount of pDNA decreased, from 40.5 ± 1.1% GFP^+^ cells at 1 μg pDNA/well to 69.4 ± 1.3% GFP^+^ cells at 0.1 μg/well. No further increases in transfection efficiency were observed at lower pDNA concentrations.

**Figure 4:**
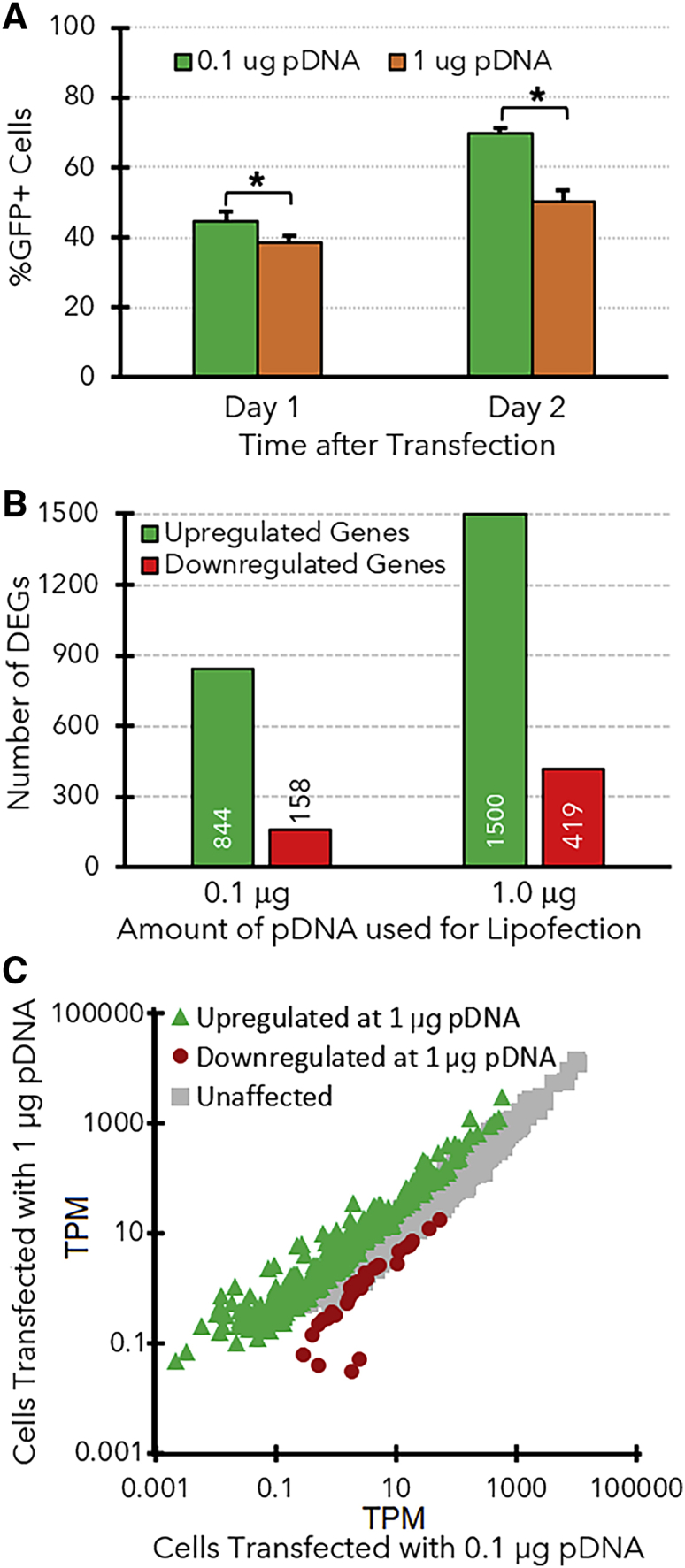
Effects of pDNA amounts during transfection on the PC-3 transcriptome (A) Transfection efficiencies obtained with PC-3 cells using lipofectamine (2.75 μL/well) and pDNA (0.1 or 1.0 μg), measured at either 1 or 2 days after transfection. Error bars indicate the standard deviation from each triplicate of samples. (B) A summary of the number of genes that were upregulated (green bars) or downregulated (red bars) after each type of transfection. ∗Significant differences in transfection efficiency. (C) Gene expression levels (TPM) for PC-3 cells that were lipofected with either 0.1 or 1.0 μg pDNA. Green triangles indicate genes that were significantly upregulated in the cells that received the higher dose of pDNA, while red circles indicate genes that were downregulated in those cells.

We next compared the transcriptomes of PC-3 cells that were transfected with either 1 μg pDNA/well (the amount recommended by the manufacturer of lipofectamine LTX) or the lower amount of 0.1 μg pDNA/well that provided the maximum transfection efficiency. The cells transfected with the lower amount of pDNA exhibited a dampened innate immune response (n = 844 DEGs) compared to the samples that received the 10-fold higher amount of pDNA (Figure 4B). A direct comparison of the transfected samples also identified 370 genes with expression levels that decreased concomitantly with the dose of pDNA, including several inflammatory cytokines (Figure 4C, Table S5). For example, while IFNλ1 was upregulated in both transfected samples, its TPM decreased 5-fold between the cells treated with 1 μg or 0.1 μg pDNA. In contrast, the expression levels of most of the CSGs with known antiviral functions were not significantly affected (e.g., IFITM1, ISG20, etc.) (Table S6) by the amount of transfected pDNA. Nonetheless, there were several other genes with TPMs that significantly decreased when the cells were transfected with less pDNA (Table S7).

### Overexpression of IFI16 in HEK-293T cells

**Figure 5:**
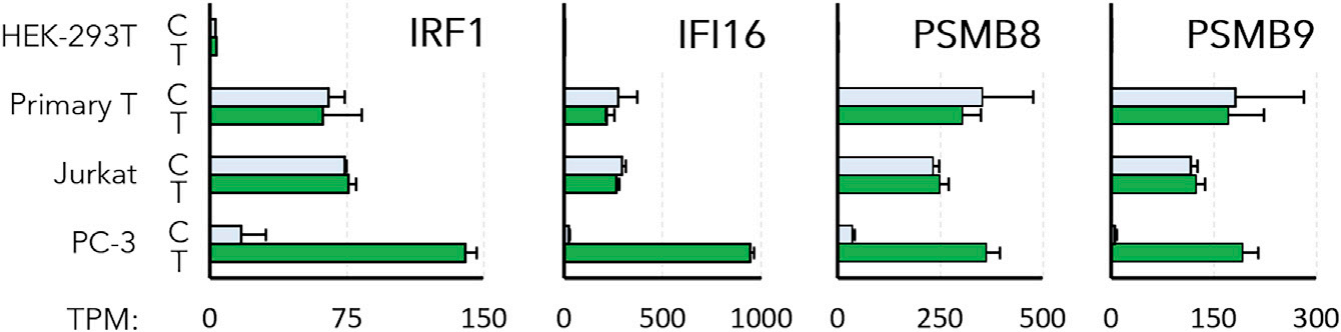
Potential antagonists of transgene expression Expression levels (TPM) of four representative CSGs that were observed to be absent or expressed at low levels in HEK-293T cells, upregulated during lipofection in PC-3 cells, and constitutively expressed at relatively high levels in Jurkat and primary T cell lines. Error brs indicate the standard deviation from each triplicate.

**Figure 6:**
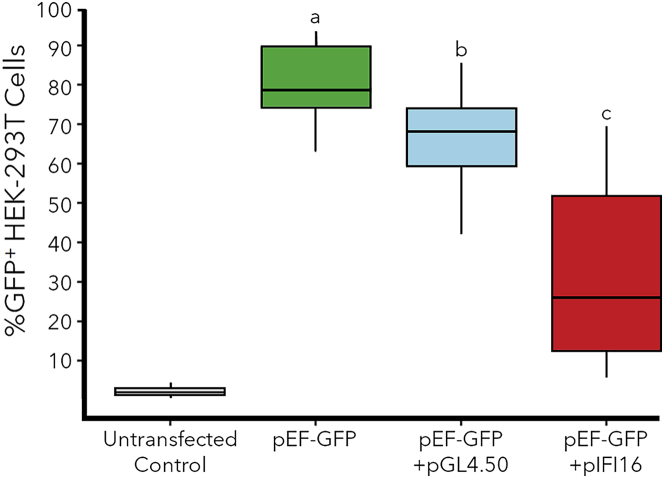
Effects of IFI16 overexpression on pEF-GFP transfection efficiency (%GFP^+^ cells) in HEK-293T cells Untransfected negative control cells were not transfected, while positive control cells were only transfected with pEF-GFP (green box). The blue box represents cells that were transfected with the luciferase expression plasmid pGL4.50 one day prior to transfection with pEF-GFP, while the red box represents cells that were transfected with the IFI16 expression plasmid pIFI16-FL one day before transfection. Horizontal lines within boxes represent the means for each group, while boxes show the interquartile range, and the entire dataset is contained within the whiskers. Letters (a, b, c) indicate samples with significantly different transfection efficiencies (n = 18 for each sample, p < 0.05 using a Wilcoxon rank-sum test).

Our experiments in PC-3 cells demonstrated that cells with a lower number of DEGs tended to have a greater transfection efficiency, so we next sought to identify specific DEGs (e.g., cytokines, CSGs, etc.) that might be responsible for this trend. Specifically, we hypothesized that resistance to transfection may be conferred by genes that are highly expressed in cells with low transfection efficiency and expressed at low levels in cells or conditions that provide greater transfection efficiency. For example, analysis of the gene expression patterns in the different cell lines identified several genes (e.g., IFI16, IRF1, PSMB8, and PSMB9) with expression levels that seemed to be inversely correlated with the transfection efficiencies of the host cell lines (Figure 5). Specifically, IFI16 was absent in HEK-293T cells, significantly upregulated in PC-3 cells, and constitutively expressed at high levels in both transfected and untransfected T cells. Furthermore, IFI16 was also expressed at a significantly lower level in the PC-3 cells that were electroporated (TPM = 149) or lipofected in SCM (TPM = 421), relative to the PC-3 cells that were lipofected in SFM (TPM = 945) and demonstrated a lower transfection efficiency. To test the hypothesis that genes like IFI16 may interfere with transgene expression, IFI16 was transiently overexpressed from a plasmid (pIFI16) in HEK-293T cells, then those cells were subsequently transfected with pEF-GFP on the following day. Figure 6 shows that the HEK-293T cells that were only transfected with pEF-GFP achieved a high transfection efficiency (80.3% GFP^+^ cells), while cells that were co-transfected with pIFI16 and pEF-GFP exhibited a significantly lower transfection efficiency (33.2% GFP^+^ cells). To determine if this lower transfection efficiency was due to the dual transfections, parallel control cultures were co-transfected with the luciferase expression plasmid pGL4.50 and pEF-GFP using the same protocol. Cells in this cohort did exhibit a significantly lower transfection efficiency (65.5% GFP^+^ cells) than the cells transfected with only pEF-GFP, but a Wilcoxon rank-sum test showed a significant decrease in transfection efficiency between the pGL4.50 group and the pIFI16 group (p = 2 × 10^−5^).

## Discussion

### Differences in DNA-sensing pathways

One of the most interesting observations in Figure 2 is the low number of upregulated genes in the HEK-293T cells (n = 142). In addition, while several inflammatory cytokines (e.g., IFNβ, IFNλ, and IL-6) and CSGs were upregulated in the transfected PC-3 cells, no cytokines or CSGs were detected in the HEK-293T cells (Table S2). This dampened innate immune response suggests that HEK-293T cells may be missing one or more components of the DNA sensing pathways that drive the potent innate immune response in PC-3 cells.

Indeed, multiple components of the CpG-DNA-sensing pathways were not detected in HEK-293T cells (Table 1), including the endosomal CpG-DNA sensor TLR9, which was also absent in PC-3, Jurkat, and primary T cells. The cytosolic CpG-DNA sensors DHX9 and DHX36 were detected in all the cell lines, but the downstream adaptor protein MyD88 that is required for signaling by DHX9 and DHX36 (Figure 1) was expressed at a relatively low level in HEK-293T cells (TPM = 2.0 vs. TPM = 298.4 in PC-3 cells).^38^ Therefore, while CpG-DNA sensing by DHX9 and DHX36 might contribute to the innate immune response to pDNA in PC-3 cells, the lack of MyD88 in HEK-293T cells may prevent them from sensing foreign CpG DNA.

**Table 1:** Expression levels (TPM) of genes involved in DNA sensing pathways in each cell line.

		HEK-293T		PC-3		Jurkat T		Primary T	
		Ctrl	*trans*	Ctrl	*trans*	Ctrl	*trans*	Ctrl	*trans*
PYHIN sensors	IFI16	0	0	25.7	945.5	294.1	266.8	274.9	215.1
	PYHIN1	0	0	0	0.1	1.1	0.9	19.6	10.2
	MNDA	0	0	0	0	0	0	0	0
AIM2	AIM2	0	0	0	10.0	0	0	28.8	34.1
	PYCARD	0.1	0.1	6.1	6.8	0	0	63.0	22.9
	CASP1	0	0	0	56.0	0	0	29.9	24.0
LRR-FIP1	LRRFIP1	39.1	45.0	135.3	129.7	105.3	96.4	168.2	159.8
	CTNNB1	225.4	311.2	269.0	195.8	105.6	99.0	105.5	103.8
	TCF4	5.5	6.9	10.5	21.2	2.6	2.3	0.7	0.5
STING-axis DNA-sensing pathway	CGAS	0.1	0.2	25.2	46.5	0	0	42.0	49.8
	DDX41	58.6	63.0	123.0	91.6	113.2	121.3	73.2	77.3
	ZBP1	0.1	0.1	0	5.8	0	0	1.3	0.2
	POLR3A	54.9	52.9	97.5	107.3	30.0	29.0	18.0	19.1
	DDX58	2.7	2.3	17.8	541.2	11.3	10.1	9.6	7.0
	MAVS	13.9	18.2	46.0	23.6	27.0	26.4	30.7	25.0
	STING1	0.2	0.6	19.0	42.2	56.6	59.9	144.1	141.7
	TBK1	44.1	38.1	40.8	39.9	30.8	25.7	54.0	51.7
MYD88-axis DNA-sensing pathway	TLR9	0.1	0.1	0.1	0.4	0	0	0.9	0.4
	DHX9	260.9	288.0	133.0	85.6	341.0	311.8	198.7	222.8
	DHX36	63.8	65.3	101.1	94.3	88.8	73.9	51.5	53.0
	MYD88	1.3	1.9	52.9	298.3	77.8	84.3	60.9	48.4
	IRAK1	171.5	195.1	163.9	147.2	158.5	162.0	113.1	135.7
	IRAK4	8.9	8.5	13.4	14.3	24.0	21.1	18.1	14.9
	TRAF6	4.0	4.8	8.0	9.6	7.1	6.8	6.0	6.1
	MAP3K7	34.6	36.1	37.3	22.1	51.8	44.8	29.7	26.8
	TAB1	36.7	40.3	18.2	22.4	26.4	28.7	21.9	17.0
	TAB2	63.5	65.7	47.4	61.0	33.8	30.0	117.7	113.2
	MAP2K4	34.8	40.6	28.4	18.5	26.4	24.5	24.4	26.8
	MAP2K7	54.9	54.3	36.6	41.3	39.4	42.2	39.6	38.3
	MAPK8	20.2	20.8	21.5	18.1	102.1	89.7	22.4	24.1
	MAPK9	57.4	55.3	39.9	21.8	47.1	44.1	29.3	27.2
	MAP2K3	31.6	39.7	46.6	74.4	53.6	55.1	178.0	170.9
	MAP2K6	6.8	14.9	41.5	19.4	17.0	14.6	4.5	1.8
	MAPK14	42.2	40.7	44.3	28.0	83.5	77.7	41.7	34.7
	JUN	104.4	154.1	47.6	159.7	6.4	5.7	144.4	138.7
	MAP3K14	6.5	5.5	14.4	25.3	5.0	5.5	38.6	34.3
	IKBKE	0.4	0.5	9.7	18.6	18.9	20.0	16.0	9.0
	NFκBIA	16.9	18.8	67.5	431.7	32.2	31.5	394.7	525.5
Transc. factors	NF-κB	18.7	20.9	34.7	44.0	52.5	48.2	97.5	103.9
	IRF5	0	0	2.9	5.4	0.6	0.6	36.1	30.1
	IRF7	0.8	1.1	23.7	379.1	5.7	6.1	26.1	27.4
	IRF3	65.5	71.5	63.5	79.8	76.3	81.5	92.2	82.2

The STING axis is an alternative cytosolic DNA-sensing pathway that may also drive the innate immune response to pDNA in PC-3 cells. PC-3 cells express each component of the STING-mediated DNA-sensing pathway (Table 1), which is induced when IFI16 or cGAS binds to cytosolic dsDNA and then signals through STING to activate TBK-1, IRF3, and nuclear factor κB (NF-κB) (Figure 1). In contrast, several important components of this pathway were either absent or expressed at relatively low levels in HEK-293T cells. For example, IFI16 was detected in PC-3 cells and upregulated 37-fold after lipofection, but it was absent in HEK-293T cells. Three more components of the STING pathway (cGAS, STING, and IKBKE) were also upregulated in the transfected PC-3 cells, but expressed at negligible levels (TPM = 0.1–0.6) in HEK-293T cells. Altogether, these data show that while PC-3 cells can sense pDNA with multiple redundant pathways and mount a potent innate immune response that induces hundreds of cytokines and CSGs, HEK-293T cells lack essential proteins at bottlenecks in DNA sensing pathways (e.g., MyD88 and STING), which may explain the lack of cytokine and CSG expression observed in HEK-293T cells (see Tables S2 and S3, respectively).

It is worth noting that 142 genes were upregulated in the transfected HEK-293T cells, but those genes do not have any known functions that could potentially inhibit transgene expression. Instead, it is possible that those genes may have been upregulated in response to the stress or toxicity caused by the presence of lipofectamine.

Many other groups have also observed the high transfection efficiency exhibited by HEK-293T cells, which has led to their widespread adoption in industry and academia.^33^^,^^39^^,^^40^ The high transfection efficiency and lack of CSG expression in HEK-293T cells may be due to the SV40 large T antigen, which was integrated into the parental HEK-293 cell genome to create the HEK-293T cell line.^31^ The SV40 large T antigen has previously been shown to improve the replication efficiency of DNA viruses^32^ and inhibit the induction of IFN expression by the cGAS-STING signaling pathway in other cell lines.^33^ Likewise, proteins expressed by the human papilloma virus have also been shown to inhibit STING,^41^ while the absence of STING in hepatocytes has been correlated with a lack of cytokine expression during hepatitis B virus infection.^42^ Regarding nonviral gene delivery, the inhibition of STING with small molecule inhibitors (C176 and C178) has been shown to enhance transgene expression by up to 3-fold in a variety of cell lines, including primary T cells.^43^ These previous studies and our observations collectively emphasize the importance of the STING DNA-sensing pathway and suggest that targeting STING for inhibition may be an effective strategy to improve the potency of gene therapy treatments.

### Downregulated genes in PC-3 cells

A large number of genes (776) were significantly downregulated in PC-3 cells after lipofection. For example, some actin (ACTG2, ACTA1), actin-related (ACTR3B, ACTR2, PHACTR3), and myosin (MYO1D, MYO5A) genes were downregulated up to 12-fold (e.g., ACTA1). This inhibition of endocytic transport genes could hamper the nuclear delivery of lipoplexes, which depends on these mechanisms.^44^ Another gene that was downregulated 5-fold in PC-3 cells was SMAD6, which has been shown to potently inhibit activation of the innate immune response.^45^ Specifically, SMAD6 activates the transforming growth factor-β pathway, which slows the activation of NF-κB and the innate immune response via degradation of MyD88.^46^ Altogether, these results show that downregulation of some host cell genes may limit the transfection efficiency of PC-3 cells.

### Constitutive expression of CSGs in T cells

The Jurkat and primary CD3^+^ T cells showed almost no response to lipofection, except for the upregulation of a few metallothioneins (MT) (MT1H, MT1E, and MT1F). MTs have previously been shown to restrict bacterial and viral replication, but it is unclear how they may interfere with nonviral gene delivery or expression.^47^^,^^48^

In contrast with the PC-3 cells, no IFNs or CSGs were upregulated after lipofection in either T cell line (Table S2). It is worth noting that some chemokines (CXCL8/10/13) and TNF-α were detected in the primary T cells, but each of these targets was detected in both the transfected and untransfected cells. Therefore, it is more likely that these cytokines were induced by the activation of the primary T cells with IL-2 rather than the transfection of pDNA.

The lack of IFN and CSG upregulation after lipofection of the T cells is somewhat surprising, since transcripts for all the requisite components of multiple DNA sensing pathways were detected in the T cells (e.g., IFI16, STING, TBK-1, and IRF3). Therefore, the T cells should be able to induce expression of cytokines, but interleukins and interferonss like IFNλ1 were not expressed in the T cells after transfection (Table S2). Similar observations were made in a previous study that also detected IFI16 and TBK-1 expression in T cells, but no IFN expression.^49^ Therefore, it seems that T cells may either lack an unknown component that is required for cytokine induction or the IFN genes may be epigenetically silenced.^50^

Although cytokines and CSGs were not upregulated in T cells after lipofection, several CSGs with established antiviral activities were constitutively expressed in both transfected and untransfected T cells. Furthermore, many of the CSGs that were expressed in the T cells were completely absent in the easily transfected HEK-293T cells and significantly upregulated in the PC-3 cells that had a moderate transfection efficiency. This inverse correlation between the expression levels of these CSGs and the transfection efficiency of each host cell line suggests that these specific CSGs may inhibit transgene uptake or expression. A complete list of the CSGs which follow this trend (absent in HEK-293T, upregulated in PC-3, and constitutively expressed in T cells) is shown in Table S3.

While Table S3 consists of a wide variety of 54 different CSGs, the four genes highlighted in Figure 5 show the strongest correlations between expression levels and transfection efficiencies. For example, the constitutive expression of CSGs in the absence of IFNs may be driven by the transcription factor IRF1, which was upregulated 8-fold in PC-3 cells after lipofection and detected in all T cell samples (TPM = 62–76). Unlike other IFN regulatory factors (IRFs), IRF1 does not require phosphorylation to activate its target genes. Therefore, IRF1 may continuously drive a high level expression of CSGs in T cells.^51^

IFI16 is another important antiviral restriction factor. As previously mentioned, IFI16 is a cytosolic DNA sensor, but recent studies have revealed additional antiviral functions for IFI16.^52^ For example, IFI16 can repress viral genes by directly binding viral promoters to inhibit transcription.^27^^,^^53^^,^^54^ Some viruses have adapted to this problem by expressing proteins (e.g., ICP0 from herpes simplex virus 1) that induce the degradation of IFI16 to prevent its transcriptional repression.^25^ Therefore, while IFI16 is unable to trigger the expression of IFNs and other cytokines in T cells, IFI16 may instead repress the transcription of both viral and nonviral transgenes by binding to their upstream promoters. Additionally, IFI16 is known to form an inflammasome complex with PYCARD, caspase 1/8, and gasdermin D upon binding to foreign DNA, which can lead to inflammation and cell death. Indeed, pyroptosis has been observed during abortive HIV infection of T cells.^55^^,^^56^ Figure 6 also shows that overexpression of IFI16 in HEK-239T cells subsequently decreases GFP expression. This decrease in transfection efficiency is most likely not due to the DNA sensing activity of IFI16 since HEK-293T cells lack STING and TBK-1. In fact, it has been mechanistically determined via an ISRE-luciferase reporter that overexpressing IFI16 alone cannot induce an IFN response or activate the STING pathway in HEK-293T cells without the simultaneous overexpression of caspase-1 and ASC.^57^ Therefore, the decrease in transfection efficiency caused by expression of IFI16 alone can more likely be attributed to IFI16 directly binding to the transgene and repressing its transcription, a phenomenon previously described for other viral transgenes.^27^^,^^53^^,^^54^

Several of the CSGs listed in Table S3 have very well-established roles in the adaptive and innate immune responses to viral infection, but it is unclear how they might inhibit the expression of nonviral transgenes. For example, PSMB8 and PSMB9 are two closely related CSGs that were upregulated in PC-3 cells and expressed at similarly high levels in both T cell lines. PSMB8 and PSMB9 have been shown to regulate transcription during the innate immune response, but they are more widely known for their role in the assembly and function of the immunoproteasome, which selectively degrades foreign proteins into peptide antigens that are then displayed on MHC-1.^58^^,^^59^^,^^60^ This process is essential to the recruitment of cytotoxic CD8^+^ T cells to virus-infected cells *in vivo*, but it is not yet known if the immunoproteasome may specifically interfere with transgene expression.

Finally, in addition to the innate immune response and CSGs, it is also important to highlight a few other differences in the phenotype and expression levels some important genes in the T cells that may affect transfection efficiency. For example, HEK-293T and PC-3 are adherent cell lines that express high levels of syndecans (SDCs) and heparin sulfate proteoglycans (HSPGs) that enable attachment to the extracellular matrix and facilitate endocytosis. In contrast, Jurkat and primary T cells grow in suspension and exhibit a notable lack of HSPGs and SDCs that may hinder their uptake of lipoplexes (see Table S4).^61^

### CSGs that modify membrane composition

In addition to IRF1 and IFI16, mRNA sequencing revealed that other host cell genes that influence the composition of the cell membrane were also upregulated. For example, the IFITMs (IFITM1/2/3) are another group of CSGs that were heavily upregulated in PC-3 cells, expressed at a low level in HEK-293T cells and constitutively expressed at higher levels in Jurkat and primary T cells (see Table S3). The well established antiviral function of the IFITM proteins is to inhibit endocytosis and endosomal escape.^62^ Specifically, IFITM1 works to stop viral fusion directly at the cell membrane, while IFITM2 and IFITM3 alter cholesterol levels in the membranes of virus-containing endosomes, which makes them more rigid and prevents the release of viral contents into the cytosol.^63^ However, previous studies have shown that the inhibition of IFITM3 with cyclosporin H or rapamycin enhances lentiviral gene delivery.^64^^,^^65^ Since lipoplexes most likely enter the cell via endocytosis, it is possible that IFITM proteins may also prevent the release of pDNA contained in an endosome, resulting in degradation of the transfected DNA upon endosomal acidification during its maturation into a lysosome.^66^

In addition, glycerophospholipid synthesis was one of the most highly upregulated pathways in the transfected PC-3 cells. The two most prominent genes in this pathway, PLA2G4A and PLA2G4C, have been shown to play a role in changing cell membrane structure and were upregulated in approximately 6-fold in PC-3 cells.^67^ Another lipid remodeling gene, HRASLS2 (PLAAT2), was also significantly upregulated by more than 19-fold. These three genes are phospholipases that participate in the hydrolysis of phospholipids to generate narrow-tailed lysophospholipids and fatty acids. These types of lipids are derived from common phospholipids and naturally form micellar structures because their tails are narrower than their head groups. They also promote an outward membrane curvature when present in the lipid bilayer^68^ that discourages membrane fusion and endocytosis.^69^

It has been previously shown that the cGAS-STING pathway is closely linked to lipid metabolism, so the observation of these upregulated genes in the experimental data matches previously known phenomena.^70^ This was further reinforced by a lipidomics study, which revealed that the innate immune response causes restructuring of the cell membrane to include higher levels of lysophospholipids.^69^^,^^71^ Restructuring the cell membrane to include these membrane-stabilizing lysolipids could potentially inhibit transgene delivery in PC-3 cells, but further work is needed to confirm this hypothesis.

### Effects of serum and electroporation on the PC-3 transcriptome

Our initial transfections were conducted in SFM to avoid any potentially confounding effects on the transcriptome from components in the serum.^72^^,^^73^^,^^74^ However, serum components like albumin have also been reported to enhance lipofection efficiency.^75^^,^^76^ Likewise, several studies have shown that electroporation can provide relatively high transfection efficiencies, even in cell lines that are relatively difficult to transfect.^21^^,^^22^ As shown in Figure 3C, electroporation also yielded significantly higher transfection efficiencies in PC-3 cells than lipofection in SFM.

While it is possible that electroporation achieves greater transfection efficiency by simply delivering more of the transgene to the cell, there are some intriguing differences in gene expression that were observed between the lipofected and electroporated cells. For example, the TPMs of several DNA sensors, cytokines, and CSGs seemed to be inversely correlated with the transfection efficiencies of the different types of transfected PC-3 cells. Table 2 shows some of the more noteworthy genes that were highly upregulated after lipofection with SFM, but expressed at significantly lower levels in electroporated PC-3 cells.

**Table 2:** Genes expressed at significantly lower levels in electroporated PC-3 cells vs. Lipofected PC-3 cells

	Vehicle:	Lipofectamine _				Electroporation		
	Media:	SFM		SCM		SCM		
	Symbol	Ctrl	*trans*	Ctrl	*trans*	Ctrl	*trans*	Padj
DNA sensors and downstream pathways	IFI16	25.7	945.5	14.1	421.4	12.3	149.3	a
	STING1	19.0	42.2	20.1	54.1	8.9	9.1	a
	JAK2	10.8	50.3	13.0	19.3	10.5	11.7	a
	STAT2	33.8	261.9	41.0	210.7	16.3	52.8	a
	AIM2	0	10.0	0	6.6	0	2.2	b
	CASP1	0	56.0	0.5	21.8	0.6	3.8	a
Midkine, chemokines, and cytokines	MDK	599.6	2058.9	543.2	990.9	330.5	332.2	b
	LGALS9	0.07	112.23	0.08	59.30	0.21	11.36	a
	CXCL11	0.1	231.5	0	54.2	1.0	47.3	a
	CXCL10	0.1	137.2	0	48.7	0.3	29.9	a
	IFNL1	0	131.1	0	33.8	0.2	26.7	a
	IFNB1	0	113.1	0	21.0	0	16.3	a
	IFNL2	0	67.8	0.1	22.1	0.1	7.0	b
	IFNL3	0	41.2	0	16.6	0.2	5.3	b
	IFNA7	0	3.7	0	0.4	0	0	b
	IFNA10	0	1.5	0	0.1	0	0	b
	IFNA16	0	0.8	0	0.2	0	0	b
Selected CSGs	BST2	3.1	1145.8	0.6	652.8	0.1	136.3	a
	PSMB9	5.8	191.7	7.8	196.3	4.8	39.6	a
	IFIT2	23.1	3070.6	44.4	1916.0	26.4	347.8	a
	TRIM22	0.77	218.36	0.10	105.61	0.06	17.53	a
	RSAD2	2.9	1418.0	0.9	768.7	1.3	185.6	a
	LGALS3BP	84.7	1313.9	163.5	1512.9	94.9	336.8	a
	SERPINE1	90.4	789.8	285.9	1854.8	164.0	134.0	a
	CD68	50.6	727.2	88.5	439.1	55.5	109.4	a
	WARS1	98.8	704.1	119.5	256.3	99.1	129.1	a
	SAMD9	12.1	665.7	14.5	347.7	11.8	149.2	a

a Gene expression levels significantly lower in electroporated PC-3 cells versus cells lipofected in SFM and SCM.

b Gene expression levels significantly lower in electroporated PC-3 cells only compared to cells Lipofected in SFM (i.e., not significantly lower than PC-3 cells lipofected in SCM).

Several components of the STING axis of DNA sensing were expressed at significantly lower levels in the electroporated PC-3 cells. IFI16 emerged again as a notable example of this trend, since it was highly upregulated (TPM = 945) after lipofection in SFM, but expressed at significantly lower levels during lipofection in SCM (TPM = 421) and electroporation (TPM = 149). Other important genes that were expressed at lower levels in electroporated PC-3 cells include STING, mediators of cytokine signaling (JAK2 and STAT2), and components of the inflammasome pathway (AIM2 and caspase 1) that induce inflammation or apoptosis in response to cytosolic DNA.^22^ Altogether, these observations show that DNA-sensing pathways may be less active in electroporated PC-3 cells, which could explain the lower number of cytokines and CSGs observed in those samples.

Indeed, multiple chemokines and IFNs that were highly expressed in PC-3 cells after lipofection in SFM were expressed at much lower levels in the electroporated cells. Table 2 shows that all the IFNs (IFNα, IFNβ, and IFNλs) were expressed at lower levels after electroporation, along with the chemokines CXCL10/11. Similar decreases in cytokine and chemokine expression were also observed via mRNA sequencing in PC-3 cells when they were transfected with lower amounts of pDNA while keeping the amount of lipofectamine constant (Table S5). It is worth noting that decreasing the pDNA:lipofectamine ratio may result in some lipoplexes with little or no pDNA, but it has been shown that positively charged liposomes devoid of DNA can still enter cells.^77^ This means that the amount of lipofectamine delivered to the cells in these experiments should have been fairly consistent, such that the changes in the transcriptome observed in Figure 4 were due to varying the amount of pDNA delivered to the cells (but not lipofectamine). These observations suggest that delivery of an excess of pDNA to PC-3 cells may exacerbate the innate immune response (i.e., increases expression of cytokines and other genes), which can lead to an inhibition of transgene expression. Therefore, the dosing of pDNA to cells should be carefully tuned to maximize transfection efficiency while minimizing inflammation.

Another intriguing observation is that the expression of two other secreted proteins—midkine (MDK) and LGALS9—decreased slightly in the presence of serum and to much lower levels (6- to 10-fold lower) during electroporation. LGALS9 and MDK have previously been shown to inhibit the initial binding of viral capsids and cationic lipoplexes to cell membranes by blocking heparan sulfate proteoglycans (HSPGs), SDCs, and other cell surface receptors that are crucial for gene delivery.^78^^,^^79^^,^^80^^,^^81^^,^^82^ Therefore, it is possible that the lower transfection efficiencies observed in PC-3 cells cultured in SFM may be due to inhibition of lipoplex endocytosis by MDK or LGALS9.

Many other CSGs that were expressed at lower levels after electroporation than lipofection in SFM are listed in Table 2 and the spreadsheet in the supplemental information. The functions of many of these genes have not yet been revealed, but there are some CSGs with known functions that might inhibit transgene expression. For example, much like IFI16, TRIM22 can specifically bind transgene promoters to exclude other activating transcription factors (e.g., SP-1).^54^^,^^83^ Alternatively, TRIM22 has also been shown to ubiquitinate viral proteins to target them for degradation.^84^

Overall, our results emphasize the importance of DNA sensing pathways and specific CSGs (e.g., IFI16, IRF1) in the innate immune response to transgene delivery. Our findings contribute to a growing body of literature that indicate the STING axis is particularly important for DNA sensing and subsequent cytokine expression, since the expression levels of IFI16 and STING are inversely correlated with transfection efficiencies in multiple cell lines and transfection methods. Our results also show that while PC-3 cells exhibit a potent innate immune response that limits their lipofection efficiency in SFM, it is possible to increase transfection efficiency by adding serum or using electroporation to deliver pDNA.

Finally, our mRNA-sequencing experiments have identified multiple possible reasons for the notoriously low lipofection efficiencies observed for T cells in this study and previous studies, including a lack of HSPGs and the constitutive expression of repressive CSGs like IFI16 by IRF1. Several other studies have demonstrated that IFI16 can inhibit transgene delivery and expression, but additional knockout studies will be necessary to confirm the potential roles of the other CSGs in transgene expression. However, after transgene repressors have been identified, short interfering RNAs or small molecule inhibitors could be developed to inhibit these targets and potentially improve the potency of future viral and future nonviral gene therapies.

## Materials and methods

### Reagents

Lipofectamine LTX was purchased from Thermo Fisher Scientific (#15338100). The GFP and IFI16 expression plasmids were purchased from Addgene (Plasmids 11,154 and 35,051, respectively), while the luciferase expression plasmid pGL4.50 was purchased from Promega (cat# E1310).

### Cell lines

Human PC-3 prostate cancer (Cat# CRL-1435), Jurkat T lymphoma (TIB-152), and HEK-293T embryonic kidney (CRL-3216) cells were purchased from ATCC, while donated primary CD3^+^ samples of T cells from three different donors were purchased from Cellero (formerly known as Astarte Bio). PC-3, Jurkat, and HEK-293T cells were cultured in serum-containing RPMI-1640 media that was supplemented with 10% fetal bovine serum, except during the 24-h period after a transfection (unless otherwise noted). In contrast, primary T cells were cultured in serum-free X-VIVO15 media that was supplemented with 0.5 ng/mL IL-2 and anti-CD3/28 Dynabeads in a 1:1 cell:bead ratio. Dynabeads were replaced weekly, while IL-2 was added to the media every 2–3 days.

### Transfections

In preparation for mRNA sequencing, each cell line was transfected using specific conditions that maximized transfection efficiency (data not shown). Adherent cell lines (PC-3 and HEK-293T) were grown to 50%–70% confluency in T-75 flasks (approximately 5 M cells/flask) and then transfected with lipoplexes that were prepared by mixing pEF-GFP (4.5 μg) with lipofectamine LTX (9 μL) and PLUS reagent (4.5 μL) in approximately 200 μL OptiMEM media.

Jurkat T cells were seeded into T-25 flasks (2.5 × 10^6^ cells) and then transfected with lipoplexes that were prepared by mixing pEF-GFP (13.2 μg) with lipofectamine LTX (36.2 μL) and PLUS reagent (13.2 μL) in approximately 200 μL OptiMEM media. Smaller cultures of primary CD3^+^ T cells were seeded at a density of 200,000 cells/well and then transfected with lipoplexes that were prepared by mixing 1 μg pEF-GFP/well, 2.75 μL lipofectamine LTX/well, and 1 μL PLUS reagent/well.

With the exception of the lipofection and electroporation experiments conducted with SCM in PC-3 cells, the SCM was removed from the cells in all other experiments and replaced with SFM before the lipoplexes were added to each of the cell lines. Cells were then subsequently incubated for 24 h at 37°C in SFM. Finally, a Millipore Guava flow cytometer was used to quantify transfection efficiency (%GFP cells) and transgene expression (mean GFP) before RNA isolation.

### IFI16 overexpression in HEK-293T cells

HEK-293T cells were seeded into 24-well plates at 25,000 cells/well in SCM and incubated at 37°C on day 1. The wells on each plate were then divided into four groups: a negative control group that was never transfected, a positive control group that was transfected once with pEF-GFP on day 3, and two other groups that were transfected with either pGL4.50 (a luciferase expression plasmid) or pIFI16-IL (IFI16 expression plasmid) on day 2 and then transfected again with pEF-GFP on day 3. In each transfection, plasmids were administered at 1 μg DNA/well along with 1μL/well lipofectamine LTX and 0.5 μL/well PLUS reagent. Plates were incubated for an additional 48 h at 37°C and transfection efficiency (%GFP cells) was measured using a Millipore Guava flow cytometer on day 5.

### Electroporation

PC-3 cells were trypsinized, centrifuged at 1,000 RPM for 4.5 min, and then washed once with PBS before centrifuging the cells again and resuspending them in PBS again. pDNA (7.5 μg pEF-GFP) and 2.5 × 10^6^ PC-3 cells were then mixed in a total volume of 100 μL in sterile 2-mm-gap electroporation cuvettes (in that order). A Bio-Rad Gene Pulser Xcell electroporation system with the CE module was then used to briefly electroporate the cells (110 V, 25 ms, single square wave pulse). The electroporated cells were then immediately plated in preheated RPMI media with serum and incubated at 37°C until needed.

### mRNA sequencing

Total RNA samples were extracted from cell samples with a Qiagen RNEasy kit. The RNA samples (2 μg total RNA) were then submitted to either Genewiz (Jurkat samples) or the Beijing Genomics Institute (PC-3, HEK-293T, and primary T cell samples) for library preparation and mRNA sequencing. Specifically, mRNA was isolated from high quality total RNA samples with RIN of more than 9 and 28S:18S of more than 1 (measured with an Agilent 2100 Bioanalyzer) using poly-T oligonucleotide beads. The mRNAs were then cleaved into smaller fragments that were subsequently reverse transcribed by DNA polymerase I into cDNA using random N6 primers. Adapters were then ligated onto the cDNAs and the resulting libraries were sequenced using either a BGI-500 sequencer (BGI) or an Illumina HiSeq (Genewiz). Low-quality reads were then filtered to produce clean reads that were mapped to the human genome/transcriptome using HISAT, Bowtie2, and RSEM to calculate gene expression values and counts. Counts were then analyzed using DESeq2 in RStudio with independent filtering turned off (see Figure S2) to identify DEGs, which were defined as genes with adjusted p values (p_adj_) of less than 0.05 and at least a 2-fold change in TPM after transfection.

TPM values were calculated by dividing the number of counts for each gene by each gene’s length to obtain reads per kilobase (RPK) values. The RPK for each gene was then divided by the sum of the RPK values measured for all genes (and multiplied by 1 million) to obtain TPM values. The complete mRNA sequencing data (fastq files and a spreadsheet of TPM values) from these experiments are available at the NCBI GEO repository (GEO: #GSE166630).

### NGS validation with rt^2^PCR

The same RNA samples that were used for mRNA-sequencing were also analyzed with rt^2^PCR. First, mRNA was reverse transcribed using the SuperScript IV VILO™ rt^2^PCR master mix with ezDNase (Thermo Fisher #11766050) according to the manufacturer’s protocol. The resulting cDNA was then quantified using an SYBR green qPCR master mix (Thermo Fisher # 4472942) with a QuantStudio 3 qPCR instrument (Thermo Fisher). All measurements were repeated in triplicate and the housekeeping gene glyceraldehyde-3-phosphate dehydrogenase was used as a reference gene/control. The primer sequences that were used are shown in Table S1.

### Cytokine quantification with ELISA

Samples of the supernatant media were taken from PC-3 cultures at 6 and 24 h after transfection with lipofectamine and stored at −72°C until needed. DuoSet ELISA kits were then used to quantify IFNL1/3 (Biotechne #DY1598B-05) levels according to the manufacturer’s protocol.

### Statistical methods

DEGs were defined as genes having a statistically significant change in expression levels/read counts (p_adj_ < 0.05) that was at least 2-fold in magnitude over the course of three independent experiments. The p_adj_ values were calculated using DESeq2 in RStudio with independent filtering turned off (see Figure S2 for script). TPMs for each gene and cell line are shown in Figure 2 as the average of three independent trials. Significant differences in transfection efficiency between groups were determined by a Student’s t-test (p < 0.05, n ≥ 3). For the IFI16 overexpression experiment, statistically significant differences in transfection efficiencies were determined between groups using a Wilcoxon rank-sum test (p < 0.05, n = 18).

## Data Availability

The authors declare that all data supporting the findings of this study are available within the paper and its supplemental information files. All raw data are available from the corresponding author upon request. The raw data can be accessed in the NCBI GEO repository (GEO Accession #GSE166630).

## Supplemental information

Document S1. Figures S1–S4 and Tables S1–S7

Table S8. Lists of all the upregulated and downregulated genes for each type of gene delivery experiment mentioned in the manuscript, along with their corresponding padj and TPM values

Document S2. Article plus supplemental information
