# Supplementary material for: Transcriptomic analysis of the innate immune response to in vitro transfection of plasmid DNA
Source: Mol Ther Nucleic Acids. 2022 Dec 5;31:43–56. doi: 10.1016/j.omtn.2022.11.025 (PMC9800263; doi:10.1016/j.omtn.2022.11.025)
Supplement: Document S1. Figures S1–S4 and Tables S1–S7 [file mmc1.pdf]

OMTN, Volume *31*

Supplemental information

Transcriptomic analysis of the innate immune response to *in vitro* transfection of plasmid DNA

Eric Warga, Jared Anderson, Matthew Tucker, Emily Harris, and Jacob Elmer

Supplemental Information

Table S1 – Sequences of the qPCR primers used in Figure S1 and Figure S3

Target	Forward Primer	Reverse Primer
IFNB1	GAAGGAGGACGCCGCATTGA	TGCTCATGAGTTTTCCCCTGGT
IFNL1	GGTGACTTTGGTGCTAGGCT	TGAGTGACTCTTCCAAGGCG
IFNL2	GGGTGACAGCCTCAGAGTGTT	ACTCTTCTAAGGCATCTTTGGCCC
IFNL3	TGAAACTAGACATGACCGGGGAC	CGGCACTTGCAGTCCTTCAG
CXCL10	CCACGTGTTGAGATCATTGCT	TGCATCGATTTTGCTCCCCT
CXCL11	AGTCCTGGAAAAGGGCATCTG	TTTGGTCCTTTCACCCACCT
CASP1	AATACTGTCAAATTCTTCATTGCAGATAAT	AAGTCGGCAGAGATTTATCCAATAA
GAPDH	CAATGACCCCTTCATTGACC	GACAAGCTTCCCGTTCTCAG
Plasmid DNA (Amp^R gene)	TCCGGTTCCCAACGATCAAG	AGTGATAACACTGCGGCCAA

Table S2. Expression levels (TPM) of chemokines and cytokines in transfected (Trans) and untransfected (Ctrl) cells.

	HEK-293T		PC-3		Jurkat		Primary T	
	Ctrl	Trans	Ctrl	Trans	Ctrl	Trans	Ctrl	Trans
CXCL1	0.1	0.2	54.4	214.2	0	0	0.3	0.2
CXCL2	0.6	0.7	2.1	40.0	0	0	0.1	0.2
CXCL3	0.4	0.9	3.2	47.9	2.3	1.7	0.2	0.1
CXCL5	0	0.1	3.1	6.6	0	0	0	0
CXCL6	0	0.1	24.3	65.4	0	0	0.1	0
CXCL8	0.3	0.2	56.2	608.7	0	0	125.2	379.9
CXCL9	0	0.0	0	2.1	0	0	0.9	1.6
CXCL10	0	0.1	0.1	137.2	0	0	21.0	24.5
CXCL11	0	0	0.1	231.5	0	0	2.9	4.0
CXCL13	0	0	0	0.0	0	0	33.1	33.6
IFNα7	0	0.1	0	3.7	0	0	0	0
IFNα10	0	0	0	1.5	0	0	0	0
IFNα13	0	0	0.1	1.3	0	0	0	0
IFNβ1	0	0	0	113.1	0	0	0.1	0.1
IFNλ1	0	0	0	131.1	0	0	0.8	0.5
IFNλ2	0	0.1	0	67.8	0	0	0	0
IFNλ3	0	0	0	41.2	0	0	0	0
IL1β	0	0	24.2	56.9	0	0	0.6	0.3
IL6	0	0	1.2	130.0	0	0	0.4	0.5
IL18	0.1	0	91.5	143.7	0	0	0.5	0.3
TNFα	0	0	0	0.7	0	0	547.8	751.8

Table S3. Expression levels of select CSGs in transfected (Trans) and untransfected (Ctrl) cells.

Gene	HEK-239T		PC-3		Jurkat		Primary T	
Symbol	Ctrl	Trans	Ctrl	Trans	Ctrl	Trans	Ctrl	Trans
BST2	0.7	1.0	3.1	1145.8	97.3	103.7	261.8	251.8
IFI16	0.0	0.0	25.7	945.5	294.1	266.8	274.9	215.1
IFITM1	4.0	4.4	57.2	3273.8	44.1	47.1	305.2	209.1
IFITM3	6.4	4.4	476.7	3650.3	7.6	11.7	42.2	22.2
PSMB8	0.1	0.2	35.4	361.1	232.0	247.7	353.7	305.2
PSMB9	0.1	0.3	5.8	191.7	116.7	124.9	182.4	171.0
IRF1	3.0	3.5	17.1	139.7	73.7	75.8	65.1	62.1
ISG15	5.4	6.3	181.5	8355.9	161.8	166.1	61.3	63.1
OASL	0.0	0.0	9.6	2137.8	1.3	1.4	25.1	19.6
OAS2	0.0	0.0	39.1	1596.4	19.2	18.0	78.6	72.7
IL32	0.7	0.7	14.0	55.2	350.5	427.4	1613.7	1062.2
OAS1	0.0	0.0	35.0	1490.6	2.8	2.7	27.8	20.3
UBE2L6	3.0	3.3	45.5	1052.8	161.8	181.0	170.9	161.2
OAS3	0.1	0.2	53.3	1129.6	17.3	17.6	40.2	32.2
IL2RG	0.0	0.0	0.5	4.0	396.6	414.4	824.0	624.7
LAMP3	0.7	1.1	19.6	738.5	43.4	41.7	4.9	4.9
SAMD9	0.3	0.3	12.1	665.7	19.7	17.2	21.6	12.9
IFIH1	0.4	0.6	10.7	593.2	9.8	8.8	19.0	18.8
PARP9	0.9	1.4	44.2	487.8	20.9	19.2	33.9	30.8
IFI44	0.0	0.0	20.2	507.7	2.1	2.3	16.5	16.4
SP110	0.2	0.3	16.2	386.7	14.7	13.2	62.3	65.6
NMI	1.6	1.7	35.0	245.2	152.9	133.8	73.1	64.0
CD7	0.1	0.2	0.6	14.2	220.6	259.8	216.3	163.6
MYD88	1.3	1.9	52.9	298.3	77.8	84.3	60.9	48.4
ICAM1	0.0	0.7	40.5	320.4	1.5	1.5	85.7	96.8
DTX3L	0.8	1.1	33.6	329.6	30.9	28.7	31.9	26.8
GBP1	0.1	0.2	2.4	298.5	24.5	20.1	59.3	55.9
SHFL	0.6	0.6	17.9	308.3	29.5	29.9	42.6	33.9
SAMD9L	0.0	0.0	5.3	354.5	2.8	2.2	17.7	12.1
SELL	0.0	0.1	0.3	4.5	271.4	246.0	342.3	110.0
PARP14	0.0	0.0	31.7	306.9	20.6	18.5	42.6	34.7
GSDMD	0.0	0.0	72.8	245.4	59.3	62.2	45.1	37.9
LGALS9	0.9	0.8	0.1	112.2	124.7	137.1	14.5	10.1
ERAP1	2.4	3.5	36.6	130.4	60.2	55.9	71.7	63.4
STING1	0.2	0.6	19.0	42.2	56.6	59.9	144.1	141.7
TRIM14	3.9	6.5	25.6	118.7	80.3	81.6	44.5	32.4
FYB1	0.0	0.0	0.3	9.8	177.8	153.3	87.5	51.5
MVP	0.4	0.6	24.3	67.8	8.6	9.7	137.1	124.3
APOBEC3G	0.1	0.2	28.5	85.2	6.4	6.5	132.4	105.8
BIRC3	0.1	0.0	7.7	53.0	10.2	8.7	118.6	128.5
ARHGAP15	0.0	0.0	0.0	1.4	117.0	105.5	102.2	72.1
SP140L	0.0	0.1	15.4	105.9	31.7	28.9	41.2	31.0
XAF1	0.0	0.0	5.2	131.5	9.3	9.0	24.2	18.5
SLFN5	0.6	0.8	19.3	130.9	19.1	18.0	31.1	9.5
APOL6	0.0	0.0	9.9	100.4	16.7	14.5	38.4	36.9
PHF11	0.5	0.3	13.2	89.1	36.9	34.2	24.1	23.7
TRIM56	0.9	1.1	10.4	54.1	62.5	63.8	24.9	19.5
PARP12	0.0	0.0	6.9	98.6	21.2	22.8	14.0	11.0
IL7R	0.0	0.0	5.8	73.8	11.1	9.0	68.4	27.2
GIMAP2	0.0	0.0	0.0	4.7	88.8	82.6	34.0	22.6
CD96	0.1	0.1	0.5	2.3	23.9	21.5	135.0	84.9
TNFRSF14	0.0	0.1	9.4	41.4	10.4	12.3	40.7	41.7
APOL3	0.0	0.0	0.8	42.8	7.8	6.8	46.0	27.2

Table S4. Expression levels (TPM) of HSPGs in primary T cells, Jurkat T cells, PC-3 cells, and HEK293-T cells.

	HEK-293T Cells		PC-3 Cells		Jurkat T Cells		Primary T Cells	
Gene	Control	Trans.	Control	Trans.	Control	Trans.	Control	Trans.
HSPG2	2.3	2.4	41.9	34.2	0.05	0.07	0.3	0.1
SDC1	3.9	6.7	173.1	71.9	0	0	0	0
SDC2	32.4	36.0	30.3	15.1	0	0	0.1	0.1
SDC3	15.3	22.4	12.9	9.1	8.1	9.3	0	0.1
SDC4	32.5	26.0	70.6	103.4	0	0	194.1	203.7

Table S5 - Expression levels (TPM) of cytokines in PC-3 cells transfected varying amounts of pDNA (0, 0.1, or 1 μg).

Symbol	Control (0 μg pDNA)	Low (0.1 μg) pDNA	High (1 μg) pDNA
CXCL1	62.96	318.89	766.05
CXCL2	6.94	46.71	263.94
CXCL3	4.37	28.44	176.28
CXCL5	11.20	18.77*	83.92
CXCL8	45.02	526.95	2523.12
CXCL10	0.00	43.87	79.74
CXCL11	0.02	52.94	94.16
CCL20	0.14	1.71	16.50
IFNL1	0.02	24.18	122.02
IFNL2	0.00	13.40	39.47
IFNL3	0.00	14.44	39.65
IFNB1	0.04	43.35	103.22
TNFAIP3	4.23	26.72	116.87
TNFAIP6	0.02	0.70	6.58
IL6	1.14	26.37	192.81
IL24	2.47	13.97	60.43
IL11	3.02	4.04*	27.00

All listed differences in expression between samples transfected with low and high levels of pDNA respectively were determined to be statistically significant (Padj < 0.05) using DESeq2.

*Gene expression values for groups transfected with low pDNA marked with an asterisk (*) are not significantly higher than the expression level of their respective controls. All other transfected samples exhibit statistically significant levels of expression for each respective gene from their untransfected controls (Padj < 0.05, determined using DESeq2).

Table S6 - Expression levels (TPM) of cytokine-stimulated genes that were unaffected by pDNA amount.

Function	Symbol	Ctrl	Low (0.1 µg) pDNA	High (1 µg) pDNA
Inhibition of Cellular Entry	IFITM1	15.03	1539.34	1451.42
	IFITM3	511.55	2835.14	2905.17
	PLAAT2	0.53	6.23	8.40
Inflammasome	AIM2	0.01	3.38	4.95
	CASP1	0.33	8.04	12.58
	IL18*	115.14	144.58	119.23
Transcription Factors	IRF1	12.69	106.25	112.21
	IRF3*	59.10	65.93	62.78
Inhibition of Transcription	IFIT1	9.13	1218.07	968.42
	IFIT2	20.99	2270.83	2537.64
	IFI16	8.50	308.56	259.97
	TRIM22	0.06	98.18	100.10
Translational Inhibition & PTMs	ISG20	3.50	41.61	64.08
	ISG15	320.70	8907.15	9962.26
	HERC5	4.67	156.17	179.34
	UBA7	0.75	29.09	25.53
	UBE2L6	31.54	531.14	483.32
Virus-specific	RSAD2	0.34	786.79	928.29
	BST2	0.33	372.65	441.84
	LGALS9	0.00	13.33	13.89
	SHFL	13.09	187.66	147.24
	SAMD9	7.77	574.80	486.95

Gene expression levels between the samples transfected with low and high amounts of pDNA, respectively, are statistically the same for each gene (Padj < 0.05, determined using DESeq2).

Gene expression values for control groups marked with an asterisk (*) are not statistically different from the gene expression values of their respective transfected groups. All other control groups are significantly lower, meaning expression of their respective genes was upregulated by pDNA transfection (Padj < 0.05, determined using DESeq2).

Table S7 - Expression levels (TPM) of non-cytokine genes that decreased when the amount of transfected pDNA was decreased.

Function	Symbol	Control	Low (0.1 µg) pDNA	High (1 µg) pDNA
Transcription factors	CEBPB	39.99	92.68	199.69
	ATF3	5.87	65.00	355.16
	NUPR1	27.48	62.78	125.31
	MXD1	4.36	14.20	57.07
Histones	H2BU1	1.60	4.23	29.08
	H2BC8	18.84	30.79*	170.98
Stress response/ Apoptosis	GDF15	28.93	87.47	385.28
	PMAIP1	51.17	465.92	1063.84
Inhibitory proteins	CDKN1A	12.87	63.19	149.46
	INHBA	71.72	127.51*	378.40
Peroxidase	PTGS2	1.09	24.68	79.84
Protein folding	AGR2	36.42	69.26	129.65

All listed differences in expression between samples transfected with low and high levels of pDNA respectively were determined to be statistically significant (Padj < 0.05) using DESeq2.

Gene expression values for groups transfected with low pDNA marked with an asterisk (*) are not significantly higher than the expression level of their respective controls. All other transfected samples exhibit statistically significant levels of expression for each respective gene from their untransfected controls (Padj < 0.05, determined using DESeq2).

Table S8: The excel document “Table S8” includes lists of all the upregulated and downregulated genes for each type of gene delivery experiment mentioned in the manuscript, along with their corresponding padj and TPM values.

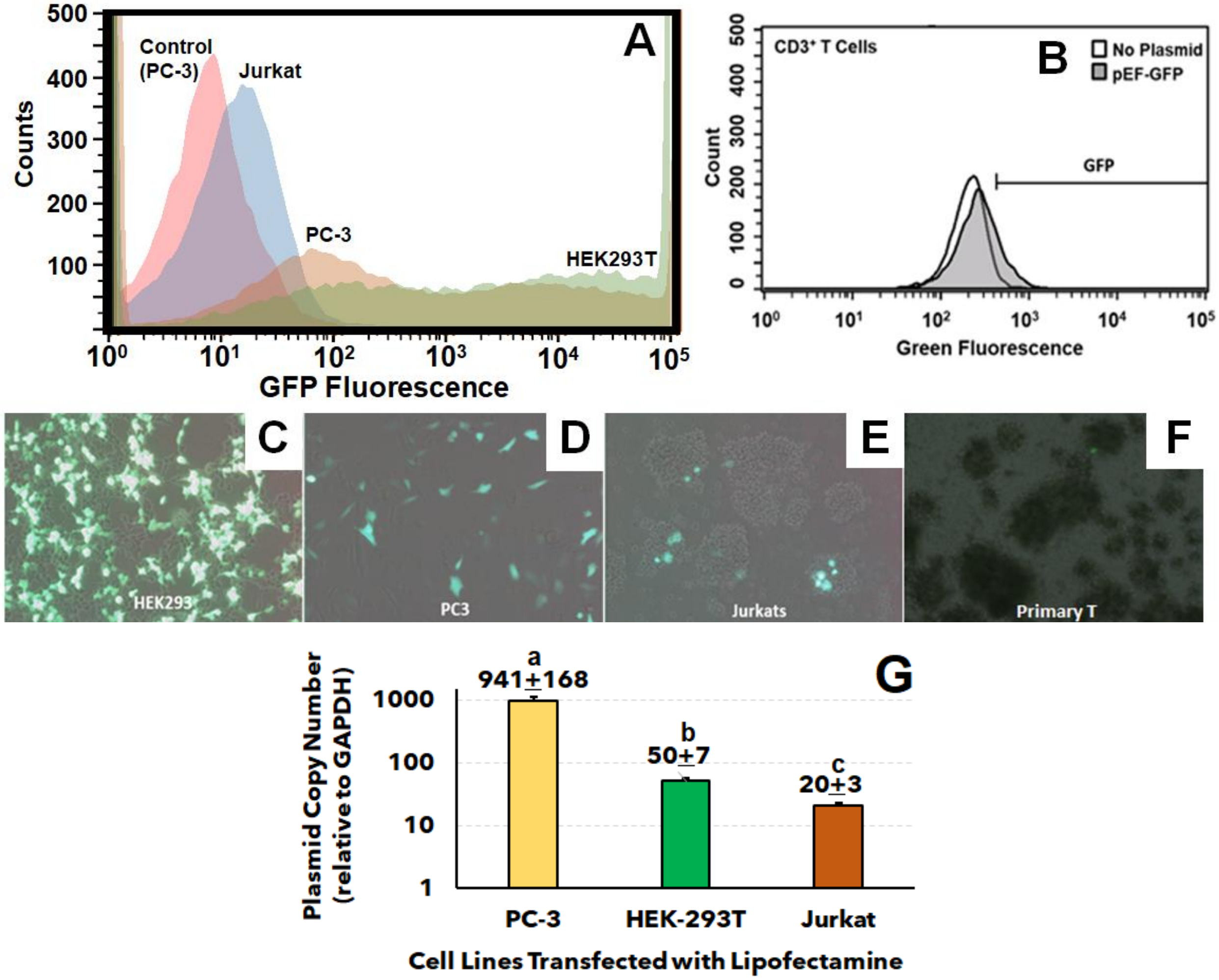

Figure S1 – Representative histograms for transfected Jurkat, PC-3, HEK-293T cells (A) and primary T cells (B) compared to untransfected control cells. (C-F) Fluorescent microscopy images of GFP expression in each cell line 24 hours after transfection. (G) Copy numbers of plasmid DNA in each cell type following transfection with Lipofectamine LTX (n = 3; letters indicate samples with significantly different copy numbers). Specifically, each cell type was seeded onto 24 well plates (6 wells of cells/plate) as described in the Methods section, then the wells on each plate were transfected with the same amounts of Lipofectamine (1.375 μL/well) and plasmid DNA (pEF-GFP, 500 ng/well). The cells were incubated for an additional 48 hours at 37°C post-transfection, then all the cells were trypsinized and a genomic DNA extraction kit was used to isolate the genomic and plasmid DNA from the cells for qPCR analysis, using SYBR Select Master Mix (LifeTechnologies, 4472942) on a QuantStudio3 qPCR instrument with the GAPDH and plasmid-specific primers shown in Table S1. The measured C_T values were then used to calculate the copy numbers of plasmid DNA (per cell) relative to the GAPDH gene using Equation 1:

$$\textbf{Eqn. 1:} \quad Plasmid\ Copy\ Number = 2^{(C_{T,GAPDH} - C_{T,Plasmid\ DNA})}/2$$

```
#Load the counts data as a matrix.  The counts file should be saved as a tab-delimited .txt file.
	cts <- as.matrix(read.csv('cts.txt',sep="\t",row.names="gene_id"))
#Load the coldata matrix, which defines which samples will be grouped and compared.  The file
should be saved as a .csv.  Columns = sample name, condition, and type.  Sample names/rows
should be the same as column names in the counts file.
	coldata <- read.csv("coldata.csv", row.names=1)
	coldata <- coldata[,c("condition","type")]
	coldata$condition <- factor(coldata$condition)
	coldata$type <- factor(coldata$type)
# Call the DESeq2 routine for analysis and build a dataset (dds)
library("DESeq2")
	dds <- DESeqDataSetFromMatrix(countData = cts, colData = coldata, design = ~
condition)
	dds
	library("DESeq2")
#Run the analysis
	dds <- DESeq(dds)
	res <- results(dds, independentFiltering = FALSE)
		res
#Order results by adjusted p-value
	resOrdered <- res[order(res$pvalue),]
#Export results as a DEG.csv file
	write.csv(as.data.frame(resOrdered), file="DEG.csv")
```

Figure S2 – Script used to analyze mRNA-sequencing counts with DESeq2 in R Studio.

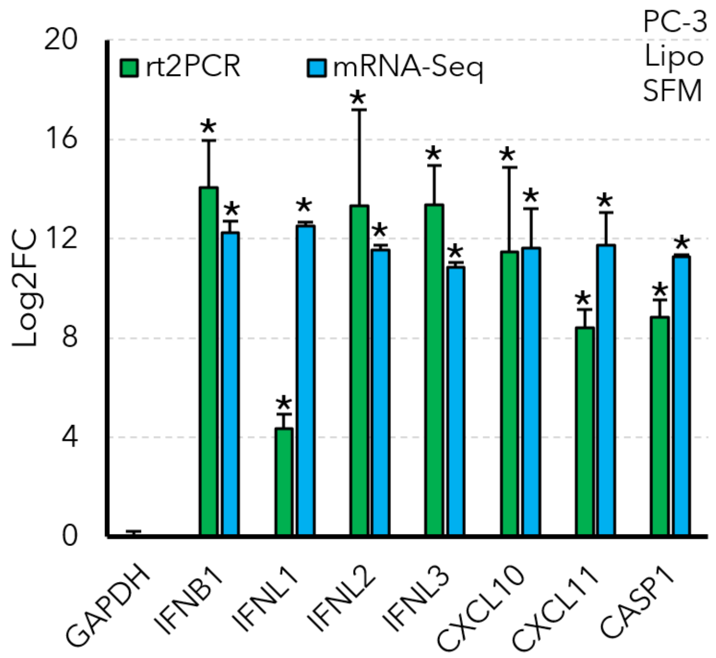

Figure S3 – Validation of mRNA-sequencing results (shown in Figure 2) with qPCR. Asterisks indicate significantly higher expression levels (log2FC) in transfected cells relative to untransfected cells ($p < 0.05$).

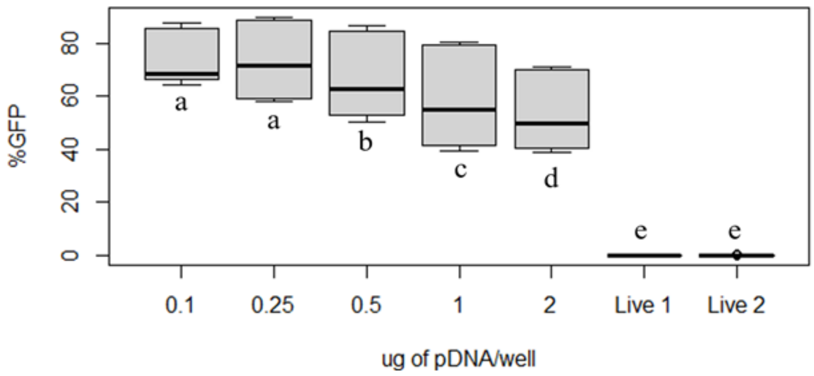

Figure S4 – Transfection efficiency in PC-3 cells with Lipofectamine over a range of pDNA concentrations (0.1-2 μg/well of PC-3 cells; Live 1 and Live 2 represent negative controls that were not transfected). Measurements of transfection efficiency (%GFP+ cells) were taken at 48 hours post-transfection. Letters indicate groups of samples with statistically significant differences in transfection efficiency that were determined using a Friedman's test ($p < 0.05$).

Methods: PC-3 cells were initially seeded at a density of 50,000 cells/well and incubated at least 24 hours at 37°C before transfections were conducted. Lipoplexes for transfection were prepared by mixing 2.75 uL of Lipofectamine with the corresponding amount of pDNA shown in Figure S3, then incubating at room temperature for 5 minutes before adding the lipoplexes to each well.
